# Zooxanthellal genetic varieties in giant clams are partially determined by species-intrinsic and growth-related characteristics

**DOI:** 10.1371/journal.pone.0172285

**Published:** 2017-02-17

**Authors:** Shota Ikeda, Hiroshi Yamashita, Shi-nobu Kondo, Ken Inoue, Shin-ya Morishima, Kazuhiko Koike

**Affiliations:** 1 Graduate School of Biosphere Science, Hiroshima University, Higashi-Hiroshima, Hiroshima, Japan; 2 Research Center for Subtropical Fisheries, Seikai National Fisheries Research Institute, Japan Fisheries Research and Education Agency, Ishigaki, Okinawa, Japan; 3 Okinawa Prefectural Fisheries Research and Extension Center, Itoman, Okinawa, Japan; National University of Singapore, SINGAPORE

## Abstract

Giant clams (tridacnine shellfishes) are large bivalves that inhabit tropical and subtropical waters and harbor the symbiotic microalgae zooxanthellae, which consist of diverse phylotypes (clades). Each clade exhibits unique physiological characteristics, and the cladal composition may influence the host's survival and its ability to tolerate environmental changes. Using quantitative PCR (qPCR) assays, we investigated the zooxanthellal genetic clades in *Tridacna crocea* (n = 93) and *Tridacna squamosa* (n = 93). These two clam species were artificially bred and maintained for an extended time period under an equivalent environment in an outdoor pond. Results showed that *T*. *crocea* had a simpler cladal composition and with an apparent dominance of clade A, whereas multiple clades were present in *T*. *squamosa*. The zooxanthellae clade A is known to occur in other zooxanthellae-bearing animals that inhabit shallow waters, which is consistent to the shallow water habitat preference of *T*. *crocea*. Interestingly, in larger individuals of *T*. *squamosa*, the main zooxanthellal clade was C rather than A. The mechanism underlying the dominance of clade C in the larger *T*. *squamosa* has not yet been clarified. However, the additional photosynthates supplied by clade C may be preferable for growing clams, as is observed in corals. The cladal composition of giant clams has previously been reported to be primarily controlled by environmental factors. However, our experiments subjected different clam species to the same environmental conditions, and our results suggested that species-intrinsic and/or growth-related processes may also influence the cladal composition.

## Introduction

Giant clams (tridacnine shellfishes) are large bivalves that inhabit tropical and subtropical seas. Currently 13 extant species of giant clams are recognized [1], and several of them, especially *Tridacna derasa* (Röding, 1798), *Tridacna gigas* (Linnaeus, 1758), and *Tridacna rosewateri* (Sirenko and Scarlato, 1991), are categorized within the Vulnerable A2cd category of the International Union of Conservation of Nature (IUCN) Red List of Threatened Species (IUCN 2016, www.iucnredlist.org. Accessed in Aug. 2016). Similar to other animals living in tropical shallow waters, such as corals, hydrozoans, and foraminifer protozoans, giant clams harbor the symbiotic microalgae zooxanthellae, particularly the dinoflagellate genus *Symbiodinium*, in their bodies [2, 3]. These clams are unique and significantly different from other *Symbiodinium*-bearing animals in terms of their method of hosting the symbiotic microalgae, where the algae do not live intracellularly but rather intercellularly in a special tubular system generated from the clams’ stomach [4]. The nutritional requirements of the giant clams are largely supplied by these symbiotic microalgae, and reports have indicated that more than 50% of the carbon requirements of these animals are supplied through the photosynthetic products (e.g., glucose and amino acids [5]) of the symbionts [6, 7]. In fact, giant clams might not need to filter-feed because the algae can provide sufficient nutrients for survival [8], although some authors have reported direct ingestion of algal cells within the clams’ stomach [9, 10].

The genus *Symbiodinium* consists of genetically diverse groups *viz* nine genetic clades (A-I) [11], with each clade consisting of finer and more diverse genetic types”. Each genetic clade or type generally possesses unique physiological characteristics [12–14] and may affect the host's survival and its ability to tolerate environmental changes (e.g., [15]). Among the nine clades, giant clams are known to exclusively harbor clades A, C, or D, or a combination of these clades [16, 17]. The composition of these clades has been previously investigated in the Indonesian populations, and the results showed that the clade composition was largely influenced by the prevailing environmental conditions, such as the seawater temperature. Namely, giant clams that hosted clades C and/or D symbionts were found in areas with higher mean temperatures, whereas others that hosted clade A symbionts inhabited areas with cooler temperatures [17]. This suggests that the symbiont structure of giant clams may be dependent on local environmental conditions. In addition, the symbiont compositions may shift as the animal grows [18]. *Acropora* corals are a well-studied group of animals, particularly with regard to their symbiosis system. They change symbiont clades according to the prevailing environmental conditions (e.g., [19]), and cladal shifts can also occur between the juvenile and adult stages of the corals [20–22]. Such symbiont shifts may be attributed to two plausible modes: “switching” and “shuffling” [23]. Switching can be an uptake of exogenous symbionts, while shuffling can be a shift from endogenous symbionts which initially resided in background levels to a major population in the hosts. Regardless of the modes, corals might select suitable or reject unsuitable *Symbiodinium* according to their life stages (e.g. [22]). Therefore, it is plausible to infer that the symbiont cladal shift in giant clams could occur because of environmental cues as well as its life stage. In the present study, we analyzed the *Symbiodinium* clades found in two species of giant clams maintained for an extended time period under an equivalent environment in an outdoor pond. The aim of the study was to clarify whether the differences in species and/or growth (size) of giant clams affected the genetic composition of the symbiont community within their bodies.

## Materials and methods

### Giant clam specimens

**Fig 1:**
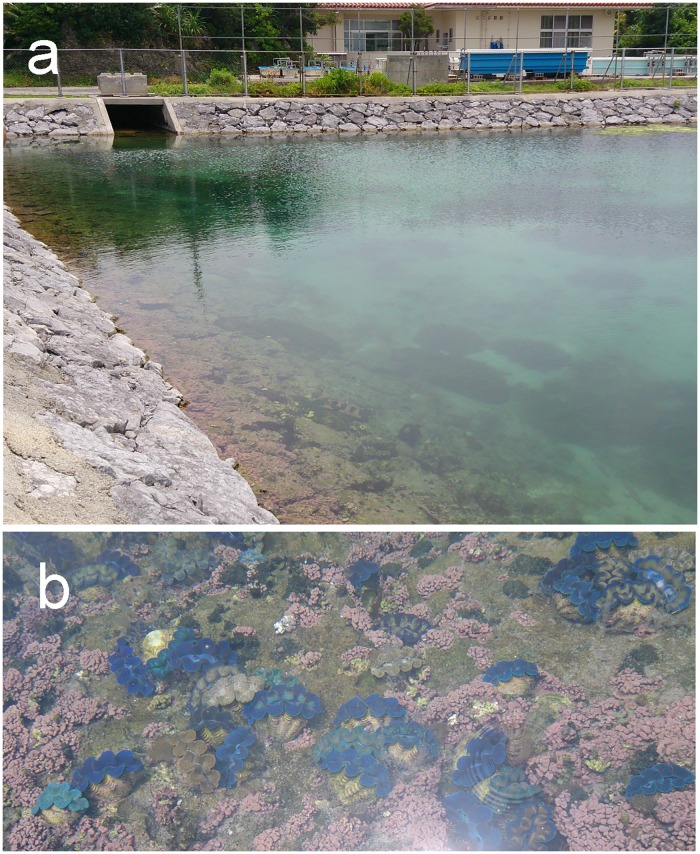
Overview of the outdoor pond (a; unlined square pond, 20 x 40 m, maximum depth = 3 m) at the Okinawa Prefectural Fisheries Research and Extension Center (Ishigaki, Okinawa, Japan) in which tridacnines were reared (b).

From June 2^nd^ to 6^th^, 2014, 96 individuals of *Tridacna crocea* Lamarck, 1819, and 96 individuals of *Tridacna squamosa* Lamarck, 1819, maintained in an outdoor pond (unlined square pond, 20 x 40 m, maximum depth = 3 m, exposed to direct sunlight; Fig 1) at the Okinawa Prefectural Fisheries Research and Extension Center (Ishigaki, Okinawa, Japan), were collected from a depth of approximately 0.5 m. The water temperature of the pond during the sampling dates were ranged from 27.3 to 27.5°C (at 0800 h). Unfortunately, environmental data were not routinely collected because the pond is primarily a sedimentation basin for the overflowing water from the fish-rearing tanks at the center. The shell lengths of *T*. *crocea* were 3.3~7.3 cm, and those of *T*. *squamosa* were 4.6~16.7 cm. The clams were artificially bred and maintained at the center and were therefore neither wild nor exposed to a natural environment. Although there was large variation in shell lengths, all the *T*. *crocea* individuals were 6 years old and all those of *T*. *squamosa* were 2.5 years old, each from a single spawning batch and thus genetically identical. In the case of *T*. *crocea*, the juveniles were derived from wild parent stocks caught by a licensed fisherman at around Ishigaki Island. The *T*. *squamosa* individuals were hybrids between a wild adult clam also caught by a licensed fisherman near the island and an adult that had been artificially bred but reared for several years at a line-off site in the Kabira Bay near the fisheries center. For initial zooxanthella infection, juveniles were exposed to ~4–10 times to zooxanthellal suspensions homogenized from adults’ mantle tissues until symbiosis was confirmed. *Tridacna crocea* symbiont sources were from the both wild stocks and artificially bred stocks reared for several years at the line-off site in the Kabira Bay. For *T*. *squamosa*, the symbiont sources were all taken from artificially bred stocks reared at the line-off site in the Kabira Bay. For typing the zooxanthella, all individuals were immediately sacrificed and a small piece (approximately 1 cm^2^) of the outer mantle was excised with scissors and a surgical knife and placed into a 1.5 ml microtube.

### DNA extraction

The mantle tissue in the tube was homogenized with a hand-held pestle homogenizer for several minutes, and then 500 μl of TE buffer (10 mM Tris-HCl, 1 mM EDTA, pH 8.0) was added to the same tube after removing the remnant tissue. The homogenate, which included *Symbiodinium* cells, was washed with TE buffer (3 times) by centrifugation (2,000 x *g*, 10 min). The pellet obtained in the final centrifugation step was then stored at −20°C until the DNA extraction. The DNA was extracted from these frozen samples by using the previously reported TE boiling method [24]. Briefly, the tubes were thawed at room temperature for 1 h and then placed in boiling water with intermittent vortexing (30 sec, 3 min intervals) for a total of 10 min.

### Clade detection

A quantitative PCR (qPCR) assay was performed because of its high sensitivity and for detection of minor clade differences. We targeted clades A, C and D based on published reports [16, 17] and preliminary trials, as only those clades were observed in all of the studied giant clams. To detect and quantify these clades, primer sets specific for the *Symbiodinium* clades (SymA28S-1F and SymA28S-1R for clade A, SymC28S-1F and SymC28S-1R for clade C, and SymD28S-1F and SymD28S-1R for clade D) were used [24]. qPCR was conducted using the prepared DNA extracts and each clade-specific PCR primer according to the protocol found in [24]. Briefly, mixtures of 1 ng μl^-1^ DNA, 0.4 pmol μl^-1^ of each forward and reverse primer, and the recommended volume of TaKaRa SYBR Premix Ex Taq II (Takara Bio Inc., Shiga, Japan), which included a ROX reference dye (as a passive reference), were analyzed in a StepOne (Applied Biosystems, Foster City, CA, USA) under the following PCR settings: 1 cycle at 95°C (30 s) and 40 cycles at 95°C (5 s) and 60°C (31 s).

### Quantification of cells from each clade

The qPCR results were expressed as the *Symbiodinium* cell numbers for each clade in the individual clams. For these quantification standards, culture strains of CS-161 (clade A; purchased from the Commonwealth Scientific & Industrial Research Organization, Australia), CCMP2466 and CCMP2556 (clades C and D respectively; purchased from the Provasoli-Guillard National Center for Culture of Marine Phytoplankton, USA), were used.

An aliquot containing 250,000 cells of each strain was trapped on a polycarbonate filter (0.8 μm pore size, 47-mm, Advantec-Toyo, Tokyo, Japan) under gentle vacuuming. The cells were frozen overnight in a 1.5 ml microtube and then thawed at room temperature with the addition of 500 μl TE buffer. The TE boiling extraction method was also employed with these filter samples according to the method described above. The resulting 2 μl of extract was equivalent to the DNA of 1,000 cells and was used as the standard. The standards were further diluted to extracts equivalent to 100, 10, 1 and 0.1 cells. Following this method, the minimum quantification range was above 0.1 cells reaction^-1^, and the clades (cells) that occurred below this minimum range were excluded from the analysis. By using these standard series, the cladal cell numbers in the giant clam samples were quantified using qPCR; however, the results are expressed as percentages of each clade because we could not standardize the mantle tissue area or volume.

### Statistical analyses

All of the statistical tests were performed using the R platform version 3.1.2 [25]. The frequencies of the *Symbiodinium* clade compositions within *T*. *crocea* and *T*. *squamosa* were analyzed using Fisher’s exact test. In this analysis, the clade compositions of *T*. *crocea* and *T*. *squamosa* were compared (see Table 1). The null hypothesis (*H*_*0*_) stated that the *Symbiodinium* clade compositions within these two species would resemble each other. To analyze the relationships between shell length (size) and *Symbiodinium* clade components, we initially determined the main clade for each individual. We used the qPCR method to quantify the cells of each *Symbiodinium* clade cells within individuals, and the main clade was defined as that with the most cells detected within it. In instances where giant clams harbored only a single clade, it was defined as the main clade. To examine whether the probability of presence of each main clade would change significantly according to the shell length of each species of clam, we performed log-likelihood ratio tests based on a generalized linear model (GLM) for each main clade. The statistical tests assumed a binomial distribution of the presence or absence of each main clade and adopted a logit-link function. In this test, the null hypothesis (*H*_*0*_) stated that each main clade would randomly appear regardless of the individual’s size. The significance probability in this test was calculated using the GLM function in R. In the present study, *p*-values less than 0.05 were considered statistically significant.

**Table 1:** *Symbiodinium* clade combinations in two tridacnine species.

	Clade combinations (number of individuals)							
Tridacnine species	A	C	D	AC	AD	CD	ACD	total
*Tridacna crocea*	61	17	0	15	0	0	0	93
*Tridacna squamosa*	10	17	0	39	0	5	22	93

### Type analysis

To identify *Symbiodinium* types rather than the clade, the DNA sequences of the internal transcribed spacer -1, -2, and nuclear 5.8S rRNA gene region (ITS region) were determined. DNA extracts from three randomly selected individuals each of *T*. *crocea* and *T*. *squamosa* were PCR amplified using ITS-rDNA specific primers for *Symbiodinium* (r18Sf and Sym28Sr) according to a previously reported protocol [26], and the products were inserted into the vector pCR4-TOPO (Invitrogen, Carlsbad, CA, USA) and cloned. Nucleotide sequences were determined using an ABI PRISM 3100 Genetic Analyzer (Applied Biosystems, Foster City, CA, USA) at the Natural Science Center for Basic Research and Development, Hiroshima University. Analyses were repeated until clone of a minor clade, which was identified based on the above qPCR result, were obtained. The sequences recovered from six randomly selected clams were initially divided to the cladal level using a BLAST search via the website of the DNA Data Bank of Japan (DDBJ; http://www.ddbj.nig.ac.jp/index-j.html). Then, each clade clone was further classified into ITS2 types according to GeoSymbio [27].

## Results

### *Symbiodinium* clade compositions within the two giant clam species

Positive qPCR results were obtained for 186 tridacnine clam individuals, 93 *T*. *crocea* and 93 *T*. *squamosa* specimens (qPCR failed for three individuals of each species), and clear differences in the trend of clade compositions were observed between these two species (Table 1). The majority of *T*. *crocea* individuals (61 of the 93 individuals) harbored only clade A, whereas *T*. *squamosa* individuals harbored multiple *Symbiodinium* clades (clades A+C, C+D, or A+C+D; 66 of the 93 individuals), and individuals harboring only clade A represented approximately 10% of the sample. Clade compositions differed significantly between the two species (Fisher’s exact test: *p*<2.2e-16).

**Fig 2:**
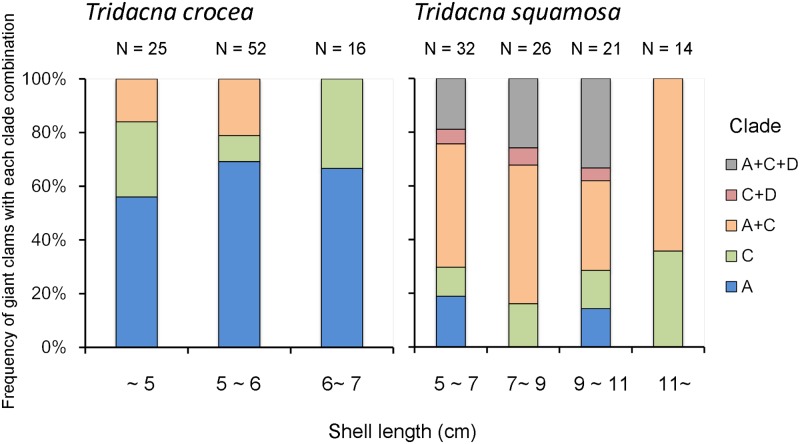
Comparison of the frequency of tridacnines harboring each combinations of *Symbiodinium* clades and the shell length (cm). N represents the number of giant clam sampled.

The frequency of giant clams with each clade combination was compared with the shell length, and these results are shown in Fig 2. In *T*. *crocea*, the most abundant shell size was 5–6 cm (n = 52), and within this size range, 69% of individuals harbored clade A only, 10% clade C only, and 21% clades A+C. Conversely, 69% of the larger *T*. *crocea* (6–7 cm; n = 16) harbored clade A only and 31% harbored clade C only. In *T*. *squamosa* (Fig 2), regardless of its shell length, individuals harboring a single clade were not the majority (n = 27), and several of the clams harbored clades A+C (n = 39). Clade D only appeared in this species in combination with other clades, and all individuals harboring clade D were less than 11 cm long. None of the clams in our sample harbored only clade D, and *T*. *squamosa* individuals with shell lengths of ≥11 cm either harbored only clade C (36%) or clades A+C (64%).

### *Symbiodinium* clade ratios within each giant clam species

**Fig 3:**
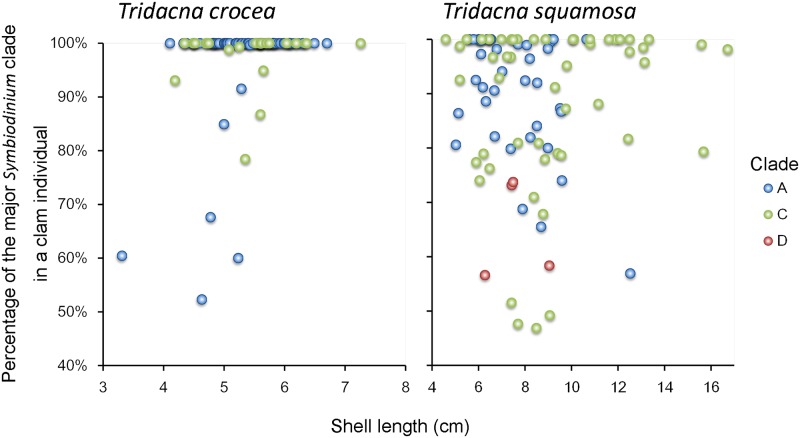
Percentage of the major *Symbiodinium* clade in a giant clam individual plotted against that shell length (cm). The dot colors indicate the dominant clade: blue = clade A; green = clade C; and red = clade D.

Next, we determined the combination of clades harbored among the all individuals as well as the ratio of clades harbored in each animal. In addition, the percentage of major clades in an individual giant clam was plotted against its shell length (Fig 3). As shown in Fig 2, most of *T*. *crocea* individuals only harbored clade A or C, with only 15 individuals harboring clades A+C. Among these 15 individuals, the number of those that harbored clade A or C as the main clade were 8 and 7, respectively. The remaining *T*. *crocea* (n = 78) harbored a single clade, A or C. Accordingly, the data for the 78 *T*. *crocea* harboring only clade A (blue) or clade C (green) are plotted on the 100% line, and the data from the other 15 individuals are plotted below it (Fig 3). Among these 15 individuals, no trend was observed regarding the dominant clade (A or C). Additionally, the presence/absence probability of clade A or clade C as the main clade was not significantly related to the shell size of *T*. *crocea* (Δdeviance = 0.00308, Δdf = 1, *p* = 0.956; and Δdeviance = 0.00308, Δdf = 1, *p* = 0.956, respectively).

**Fig 4:**
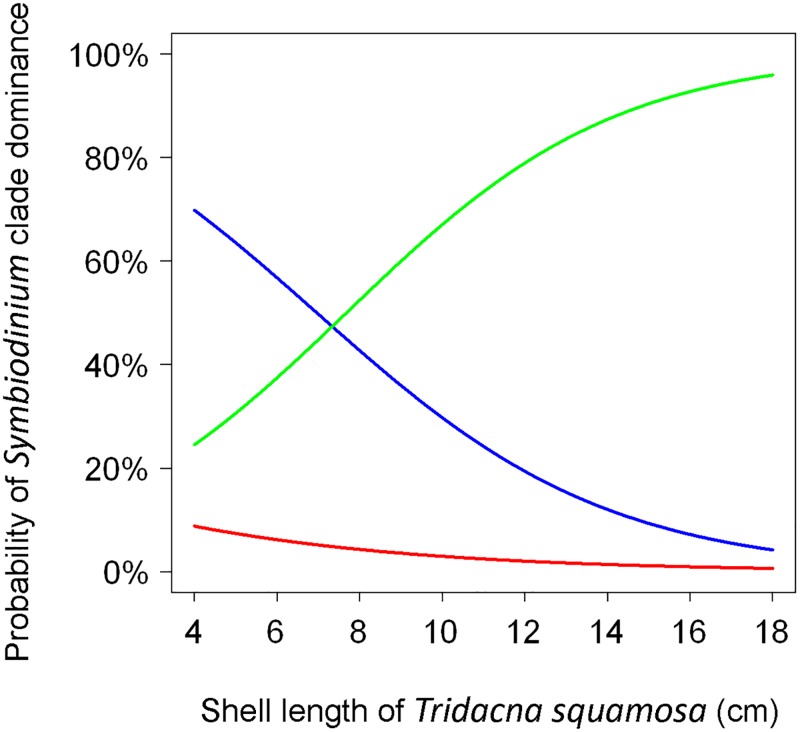
Logistic curves of the *Symbiodinium* clades in *T*. *squamosa* plotted against the shell length based on the results depicted in Fig 3. Each curve represents the probability of *Symbiodinium* clade dominance. Blue curve = clade A; green curve = clade C; and red curve = clade D.

In *T*. *squamosa*, 38, 51, and four individuals harbored clades A, C, and D as the main clade, respectively. High percentages of the plots of clade A (blue dots in Fig 3) and clade D (red dots) were primarily observed in individuals smaller than 11 cm. The presence/absence probability of clade A or clade C as the main clade was significantly related to the shell size of *T*. *squamosa*. Consequently, in the larger shell size, the number of individuals harboring clade A as the main clade significantly decreases (Δdeviance = 8.5, Δdf = 1, *p* = 0.00355), whereas the number of individuals harboring clade C as the main clade significantly increases (Δdeviance = 10.2, Δdf = 1, *p* = 0.00140). However, the presence/absence probability of clade D as the main clade was not significantly related to the shell size of *T*. *squamosa* (Δdeviance = 0.658, Δdf = 1, *p* = 0.417). Fig 4 shows the logistic curves of the decreasing probability of clade A dominance (blue line) and the increasing probability of clade C dominance (green line) according to the size of the giant clams.

### Type analysis

A total 47 clones were analyzed from six randomly selected clam samples (three for *T*. *crocea*, three for *T*. *squamosa*) (Table 2). All of the clade A sequences (32 clones) were related to type A6 or A3. Namely, 18 clones were 100% match with type A6 sequence on the GeoSymbio, and four clones were closely related to type A6 but included single nucleotide substitution (shown as A6 relative in Table 2). There are no identical sequences on the GeoSymbio, but nine clones were identical to type A3c (KR013750 on the DDBJ website), and one clone was closely related to type A3 (AF427467 on the DDBJ) with single nucleotide substitution (A3 relative in Table 2). In the clade C sequences, although only two clones were recovered, they were identical to AF195157 on the DDBJ, and this sequence closely related to type C2 and C15 group on the GeoSymbio. Remaining 13 clones belonged to clade D. The six clones were identical to type D1a on the GeoSymbio, and two clones were included single nucleotide substitution for D1a (D1a relative in Table 2). One clone was 100% match with type D5 sequence on the GeoSymbio. There was no identical sequence on the GeoSymbio, but four clones were closely related to type D5c (EU812746 on the DDBJ; D5c relative in Table 2). Except the clade D clones and the type A3 related clone (one clone), all of the types were recovered from both *T*. *crocea* and *T*. *squamosa* samples.

**Table 2:** *Symbiodinium* types retrieved from the two giant clam species (3 individuals for each species) by using PCR-cloning method.

Tridacnine species	Clade	Type	Numbers of the clones	Identical sequence for GeoSymbio or DDBJ Acc.No.
*Tridacna crocea*	A	A6	14	A6 (GeoSymbio)
		A6 relative	2	-
		A3c	6	KR013750 (DDBJ)
		A3 relative	1	AF427467 (DDBJ)
	C	C2/C15 group	1	AF195157 (DDBJ)
*Tridacna squamosa*	A	A6	4	A6 (GeoSymbio)
		A6 relative	2	-
		A3c	3	KR013750 (DDBJ)
	C	C2/C15 group	1	AF195157 (DDBJ)
	D	D1a	6	D1a (GeoSymbio)
		D1a relative	2	-
		D5	1	D5 (GeoSymbio)
		D5c relative	4	-

## Discussion

In this study, we performed a quantitative PCR (qPCR) assay to detect *Symbiodinium* clade within giant clams, and our work represents one of the first investigations. Various methods have been developed for genetic identification of *Symbiodinium*, including DNA cloning, DGGE (denaturing gradient gel electrophoresis), TGGE (temperature gradient gel electrophoresis), and SSCP (single-strand conformational polymorphism). However, these methods often neglect the minor occurrences of *Symbiodinium* (ca. 5–10%) [28, 29], especially in the case of clade D [30]. In contrast, qPCR method used in this study can detect minor compositions at a level of 0.1–0.01% [24]. Thus, by employing this method, minor clades sometimes occurring in background levels can also be detected and discussed.

### Type and clade compositions

The majorities of specimens of each giant clam species possessed clade A (81.7% of *T*. *crocea* and 76.3% of *T*. *squamosa*) and shared the closely related type A3/A6 group, although these types were not quantitatively analyzed. These types are now identified as *Symbiodinium tridacnidorum*, and as indicated by the species name, this species is recognized as associating with tridacnine clams [31]. For other clades, 34.4% of *T*. *crocea* and 89.2% of *T*. *squamosa* contained clade C, and we recovered the sequences closely related to type C2 / C15 group from the both species. This type group has been known to associate with various species of tridacnines (e.g. [16, 17]). Clade D was found only with *T*. *squamosa*. The types D1a and D5 groups are also known to associate with various species of giant clams (e.g. [17]) as well as with various corals.

Although type quantification was not achieved, clade composition or clade dominance trends differed significantly between *T*. *crocea* and *T*. *squamosa*. Although the two species were maintained within the same pond under an equivalent environment, *T*. *squamosa* harbored clades A, C and D, whereas *T*. *crocea* lacked clade D and was most commonly associated with clade A. Generally, *T*. *crocea* tends to inhabit shallower depths than other species of clams [17]. Winters et al [32] reported that stony coral *Stylophora pistillata* individuals inhabiting shallower waters are more closely associated with clade A, whereas *S*. *pistillata* individuals in deeper waters are more closely associated with clade C. These authors found that *S*. *pistillata* associated with clade A showed less susceptibility in a maximum photosynthetic yield (*Fv/Fm*) than the individuals with clade C under thermal stress. Clade A *Symbiodinium* has also been found in other corals that inhabit shallow, high-irradiance waters [12]. Thus, clade A appears to be relatively insensitive to the high irradiance and high temperature stresses of shallow waters. Based on the nature of clade A, it is reasonable to suggest that *T*. *crocea*, which inhabits shallow waters, shows a closer association with clade A. However, the association with this clade may not be dependent on the environment, as *T*. *squamosa* was also raised in the same pond and did not exhibit this particular association only with clade A. Thus, there might be an intrinsic feature of *T*. *crocea* that allows it to adapt to the harsher environmental conditions of shallow waters. Conversely, *T*. *squamosa*, which is commonly found in deeper waters, was mainly associated with clade C. Moreover, *T*. *squamosa* tend to harbor more diverse symbionts than *T*. *crocea*, which is consistent with the results of a previous study [17]. However, when considering temperature tolerance, there is discrepancy between the above hypothesis and the previous findings. It has been reported that tridacnines with clade A symbionts were more susceptible to bleaching under conditions of elevated temperatures, whereas clams with clade C were less susceptible to bleaching [33]. This is consistent with the findings in Indonesian waters, where individuals harboring clade C were located in areas with a higher mean temperature, while individuals harboring clade A were more common in cooler areas [17]. Under our hypothesis, *T*. *crocea* individuals harboring clade A would be at greater risk of bleaching under elevated temperature while they inhabit shallow water. The explanation for the dominance of clade A in *T*. *crocea* remains unknown, but there might be a trade-off between temperature sensitivity and an advantage provided by clade A.

### Shell size versus clade composition

We determined the main clade within each individual, and the results showed that *Symbiodinium* clades varied depending on the shell size in *T*. *squamosa*, whereas this was not observed in *T*. *crocea*. With the increasing size of *T*. *squamosa*, the proportion of clade A among the total *Symbiodinium* population lowered, whereas the proportion of clade C became higher. Although our current investigation was a snap-shot, not tracing the same individuals over time, such cladal shifts or switching is commonly found in corals, where juveniles tend to harbor clades A, C and D initially and adults frequently harboring clade C [20–22]. Clade A has sometimes been recognized as “weed”, which is opportunistic and is relatively insensitive to stress [34]. Conversely, clade C culture strain exhibited slower growth (Yamashita and Koike. in press). Because of its tough and opportunistic nature, clade A cells were more easily isolated from tridacnines as culture strains [35]. Harboring such a “weedy” clade, i.e. clade A, might contribute to the juveniles’ environmental resilience even with the risk of bleaching under elevated temperature. However, the reason underlying the dominance of clade C in the larger *T*. *squamosa* has not been clarified. Jones and Berkelmans [36] reported that a stony coral, *Acropora millepora*, harbors clade C and exhibits faster growth than conspecifics harboring clade D under both laboratory and field conditions. Cantin et al. [37] observed an enhanced growth rate of *Acropora millepora* that was related to a greater translocation of photosynthates from clade C (C1) *Symbiodinium*. Another possible explanation for the dominance of clade C, particularly in tridacnines, is that the growth of clade C symbionts may be accelerated in the giant clam tissues. This outcome was previously observed in a culture experiment using giant clam tissue homogenates [38]. These observations along with our current findings may suggest that clade C might be advantageous for controlling the growth of the host body and acquiring the necessary nutrients. This hypothesis should be further addressed.

### Why did *T*. *squamosa* harbor the minor clade D?

It should be noted that clade D was only detected in a minor percentage of *T*. *squamosa* (less than 1%) and was limited to clams of less than 11 cm. This outcome might be explained by the hypothesis that minor background symbionts perform the role of adapting hosts to environmental shifts by shuffling the *Symbiodinium* composition to minority clades, which may be suitable for new environments [24,39–41]. Jones et al. [19] reported that a thermally sensitive type of C2, which was initially predominant in corals, shifted to clade D by symbiont shuffling after a massive coral bleaching event. Similarly, the stony coral *Pavona decussata*, which possesses clade C, is more susceptible to seasonal environmental changes than *Pavona divaricate*, which possesses clades C and D [42]. Certain corals that harbor clade D symbionts are considered to be more resistant to thermal stress compared with those that harbor clade C symbionts [43, 44]. Therefore, the presence of clade D as a minor component may be beneficial for adapting the host to unexpected environmental shifts.

## Conclusions

In the present study, we used a highly sensitive qPCR method to analyze the *Symbiodinium* clade compositions within two giant clam species (*T*. *crocea* and *T*. *squamosa*) that were maintained in the same outdoor pond. Our results demonstrated that the clade compositions differed significantly between the species. Furthermore, in the case of *T*. *squamosa*, the main dominant clade was from clade A to clade C along with increasing the clam size. DeBoer et al. [17] reported that the symbiont compositions within giant clams may be influenced by the prevailing environmental conditions, such as seawater temperature. However, the results of our study indicate that the symbiont compositions can also be affected by the clam species and size.

## Supporting information

S1 Table Shell sizes and zooxanthellal clade compositions of each *Tridacna crocea* individuals. (XLSX)

S2 Table Shell sizes and zooxanthellal clade compositions of each *Tridacna squamosa* individuals. (XLSX)
